# An improved approach for reconstructing consensus repeats from short sequence reads

**DOI:** 10.1186/s12864-018-4920-6

**Published:** 2018-08-13

**Authors:** Chong Chu, Jingwen Pei, Yufeng Wu

**Affiliations:** 1 Department of Biomedical Informatics, Harvard Medical School, 10 Shattuck Street, Boston, 02115 MA USA; 2 Department of Computer Science and Engineering, University of Connecticut, 371 Fairfield Way, Unit 2155, Storrs, 06269 CT USA

**Keywords:** Repeat elements, De novo genome assembly, Sequence analysis

## Abstract

**Background:**

Repeat elements are important components of most eukaryotic genomes. Most existing tools for repeat analysis rely either on high quality reference genomes or existing repeat libraries. Thus, it is still challenging to do repeat analysis for species with highly repetitive or complex genomes which often do not have good reference genomes or annotated repeat libraries. Recently we developed a computational method called REPdenovo that constructs consensus repeat sequences directly from short sequence reads, which outperforms an existing tool called RepARK. One major issue with REPdenovo is that it doesn’t perform well for repeats with relatively high divergence rates or low copy numbers. In this paper, we present an improved approach for constructing consensus repeats directly from short reads. Comparing with the original REPdenovo, the improved approach uses more repeat-related k-mers and improves repeat assembly quality using a consensus-based k-mer processing method.

**Results:**

We compare the performance of the new method with REPdenovo and RepARK on Human, Arabidopsis thaliana and Drosophila melanogaster short sequencing data. And the new method fully constructs more repeats in Repbase than the original REPdenovo and RepARK, especially for repeats of higher divergence rates and lower copy number. We also apply our new method on Hummingbird data which doesn’t have a known repeat library, and it constructs many repeat elements that can be validated using PacBio long reads.

**Conclusion:**

We propose an improved method for reconstructing repeat elements directly from short sequence reads. The results show that our new method can assemble more complete repeats than REPdenovo (and also RepARK). Our new approach has been implemented as part of the REPdenovo software package, which is available for download at https://github.com/Reedwarbler/REPdenovo.

## Background

A repeat is one segment of DNA that appears multiple times in the genome in identical or near-identical form. There are many types of repeats such as transposable elements (TEs), tandem repeats, satellite repeats, and simple repeats [1, 2]. Among these, TEs are perhaps the most well-known. TEs can amplify themselves in the genome using various mechanisms, typically involving RNA intermediates. TEs are believed to constitute 25% to 40% of most mammalian genomes [1, 2]. In humans, the most common TEs are Long Interspersed Elements (LINE-1s or L1s), Short Interspersed Element (SINEs), and Long Terminal Repeats (LTRs), comprising approximately 17%, 11% and 8% of the human genome, respectively. While most of the TEs in humans are inactive now, some including Alus, SVA, L1, and possibly HERV-K are believed to be still active [3].

Many computational approaches have been developed for repeat analysis. The most commonly used tools are those based on curated repeat libraries such as Repbase [4] and Dfam [5]. RepeatMasker [6] is the most widely used tool of this type. It aligns genomic sequences to known consensus repeat sequences to mask or annotate the genomic sequences. There are also tools designed for constructing repeat libraries from reference genomes. RepeatScout [7], PILER [8] and phRAIDER [9] all belong to this type. One limitation of these tools is that they all either require the reference genome or an existing repeat library to call or analyze repeats. However, for complex (e.g. highly repetitive) genomes or genomes from some recently sequenced species, there are only low quality assembled genomes available and often no existing annotated repeat libraries. Thus, it is useful to develop tools for analyzing repeats directly from short reads, without the need for either reference genomes or repeat library. RepARK [10] is one such tool. It first runs k-mer counting and isolates highly frequent k-mers. It then assembles the highly frequent k-mers to construct the repeats. RepARK has been used to construct repeats in recent publications (see e.g. [11]). One major disadvantage of RepARK is that most constructed repeats are fragmented or just pieces of the whole repeats. Recently, we developed REPdenovo [12], a computational approach for constructing repeats directly from short sequence reads. Comparing to RepARK, REPdenovo not only constructs more repeats, but also generates more complete (i.e. longer) repeats. However, REPdenovo doesn’t work well for highly divergent or low copy number repeats.

In the paper, we propose an improved method for reconstructing repeat elements from short reads. Similar to the original REPdenovo, our new method also finds and assembles these highly frequent k-mers to form consensus repeat sequences. Here are the two main improvements over the original REPdenovo:

- Our new method uses more repeat-related k-mers than the original REPdenovo for repeat assembly, and can assemble longer consensus repeats.
- Our new method runs a randomized algorithm to generate more accurate consensus k-mers than the original REPdenovo. This improves the quality of the assembled repeats.

Comparing to the original REPdenovo and RepARK, our new method can construct more fully assembled repeats in Repbase on both Human, Arabidopsis and Drosophila data, especially for higher divergent, lower copy number and longer repeats. We also apply the new method on Hummingbird data, which has no existing repeat library. Most of the repeats constructed by our new method for Hummingbird can be fully aligned to PacBio long reads. Many of these repeats are long. More than half of the Hummingbird repeats are masked by RepeatMasker, which suggests that our assembly works well. Moreover, many of the assembled repeats are likely to be novel because there are no matches in RepBase, which suggests these may be present in only Hummingbird or its close related species.

## Method

**Fig. 1:**
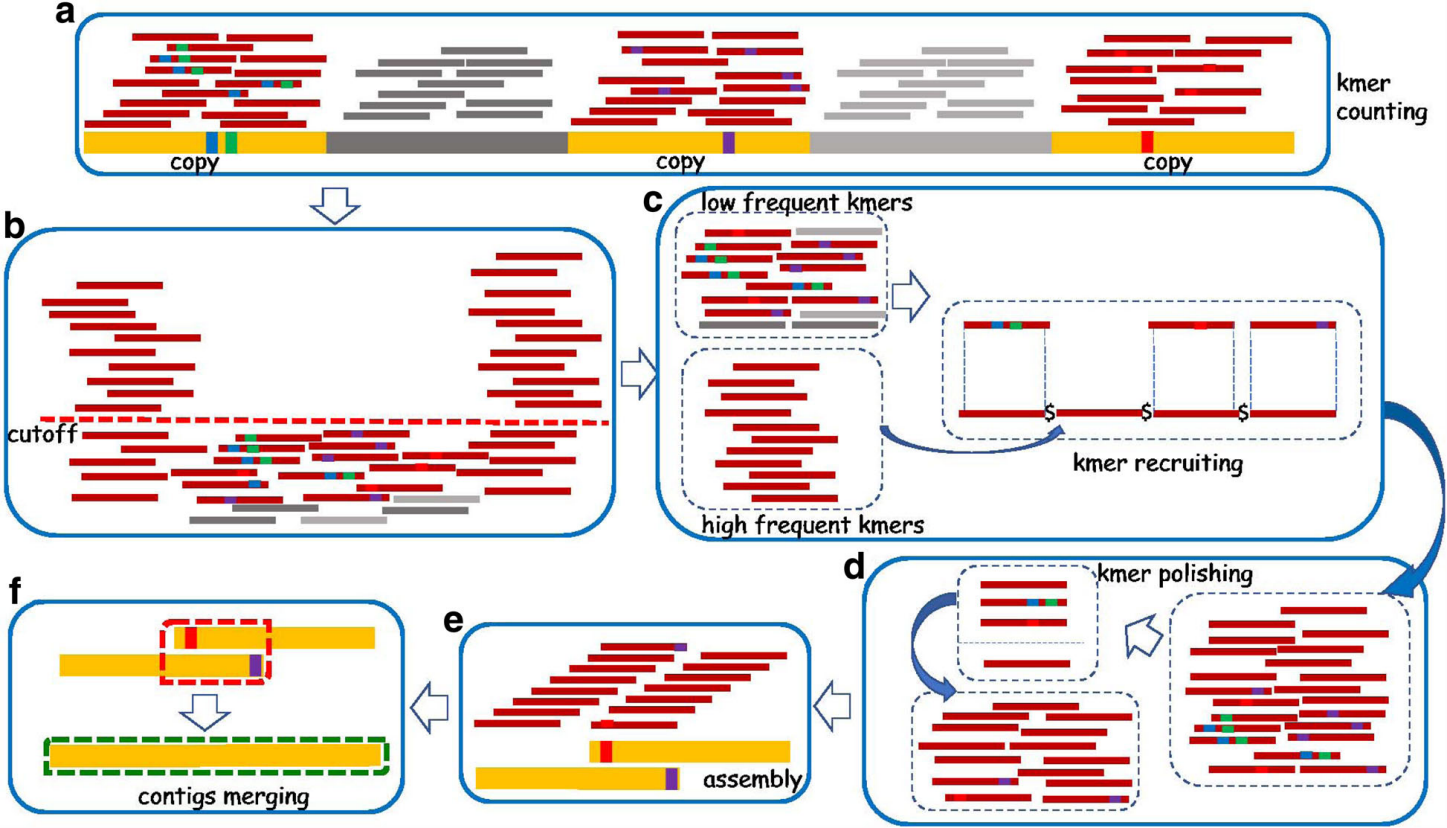
High-level procedure of improved repeat construction. Thick bars: genomic sequences. Yellow thick bars: repeat copies. Colored squares within thick bars: mutations (substitutions and indels) within repeats. Thin bars: k-mers. There are six main steps. **a** K-mer counting for the reads. **b** Find the highly frequent k-mers and k-mers with intermediate frequencies according to a user-specified cutoff on k-mer frequency. **c** Find repeat-related k-mers by aligning those k-mers of intermediate frequencies to highly frequent k-mers. **d** Improve k-mer quality with a consensus-based approach. **e** Assemble the improved k-mers. **f** Merge contigs that have reliable prefix-suffix overlap

Similar to the original REPdenovo, our new method assembles consensus repeats directly from sequence reads. The high-level procedure is shown in Fig. 1. In the following, we first provide a brief description on the repeat assembly procedure with frequent k-mers that is used by the original REPdenovo. We then illustrate the key technical problems that make the original REPdenovo perform poorly on highly divergent and low copy number repeats. We present two approaches that are implemented by our new method. These approaches allow better construction of highly divergent or low copy number repeats.

### Repeat assembly from frequent k-mers

**Fig. 2:**
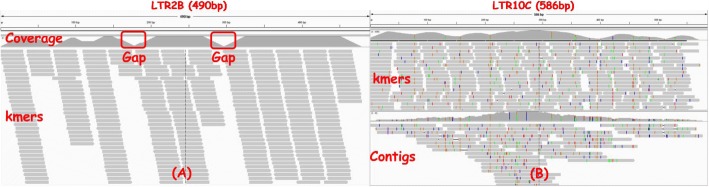
Illustration of two example repeats that are not fully constructed by the original REPdenovo. Highly frequent 30-mers of one human individual NA19230 are aligned to the human consensus repeats in Repbase. The left part (**a**) shows the alignments on repeat “LTR2B”. Two gaps are formed when 30-mers originated from highly divergent regions have low frequencies due to repeat copy divergence. The right part (**b**) shows the alignments on repeat “LTR10C”. The colored bars are variations on copies. The assembled contigs are fragmented because the 30-mers are of highly divergence

For completeness, we provide a brief introduction on repeat assembly from frequent k-mers. Repeats usually have many copies in the genome. For low divergent and high copy number repeats, k-mers generated from copies of the same repeat at the same position will be identical with high probability. Thus the frequencies of such k-mers will be higher than those of k-mers from non-repetitive regions. Thus, with given cutoff (say *n* times of the average k-mer frequency, where *n* can be viewed as the copy number), these highly frequent k-mers from repeats can be identified, while the less frequent k-mers will be discarded since they are unlikely to come from repeats. Now if we view the repeats as “genomes” and the frequent k-mers are the “reads” as in genome assembly, the repeats can be assembled from these frequent k-mers using standard genome assembly tools such as Velvet [13]. This is the key observation of RepARK and the original REPdenovo. However, in practice complete consensus repeat sequences can rarely be assembled in this way. This is because the variations on repeat copies and also read errors make the repeat copies divergent from the consensus. As the result, even for low divergent repeats, usually only short contigs can be directly assembled. Figure 2b shows one such situation. The improvement made by the original REPdenovo is that it performs a second-round assembly: it tries to assemble short contigs to form longer consensus repeats based on reliable prefix-suffix matches of the contigs. Refer to [12] for more details.

### The difficulty of assembling highly divergent repeat regions

The main problem with the original REPdenovo is that it cannot fully assemble highly divergent or low copy repeats. Even when a long repeat is overall of low divergence, there may still be regions with high divergence rate. In this case, the original REPdenovo also cannot assemble the high divergent regions within a repeat. There are two reasons. First, when the repeat divergence rate is high or the copy number is low for a region, k-mers originated from the this region will likely be of low frequency and thus are discarded. As a result, when repeat assembly is performed, only fragments of repeats will be obtained since k-mers from the highly divergent regions are missing. Moreover, even though some k-mers from highly divergent regions are present in repeat assembly, it is still challenging to assemble whole repeats. This is because contigs may break at regions with sequence variations. In Fig. 2 we show two examples to illustrate these two issues. First, we obtain highly frequent (at 10 times of the average k-mer frequency) 30-mers from real reads of one human individual NA19239. Then we align these 30-mers to the human consensus repeats released in Repbase. Two alignment cases on repeats “LTR2B” and “LTR10C” are shown in Fig. 2 through IGV [14]. The left alignment is for “LTR2B” with length 490bp. Apparently, there are two gaps with very low or no k-mer mapped, which will cause the assembly of this repeat to have at least 3 segments. The right alignment is for “LTR10C” which has more variations (the colored bars). When there are variations, genome assemblers e.g. Velvet [13] usually construct contigs that are short and fragmented.

In order to assemble repeats with higher divergence rates, we need to find more repeat-related k-mers that originate from highly divergent repeat regions. In the following, we first describe a new method for finding such less frequent repeat-related k-mers. We then use these repeat-related k-mers to improve the quality of the assembled repeats.

### Mapping-based alignment for finding more repeat-related k-mers

We now focus on assembling repeats that have higher divergences and/or lower copy numbers than those constructed by the original REPdenovo. Many k-mers from highly divergent regions may have relatively low frequencies. These k-mers are then discarded and are not included for repeat assembly. A main observation is that these discarded repeat-related k-mers usually have high sequence identity with some repeat-related k-mers with high-frequency. Recall that k-mers with high frequencies are likely to come from some repeats. Thus, if a k-mer is similar to some highly frequent k-mer, this is an indication that this k-mer is also related to some repeat. Therefore, we can compare the sequences of all the discarded k-mers with the highly frequent k-mers. If a discarded k-mer has reliable prefix-suffix match with some highly frequent k-mer, this k-mer should be kept for repeat assembly. However, direct comparison of all pairs of lower frequency and high-frequent k-mers using dynamic programming is infeasible empirically. This is because the number of lower frequency k-mers can be very large (usually in millions), and there can also be many highly frequent k-mers.

To develop a practical method, we take the following “mapping-based alignment” approach. The key idea is creating a “reference k-mer genome” by concatenating all the high-frequent k-mers. We then view the less frequent k-mers as “reads”. The reads mapping tool BWA [15] is used to align these “reads” to the reference k-mer genome. The mapped k-mers are kept for repeat assembly. This is shown in step (c) of Fig. 1. This approach works because we only want to find k-mers that have high sequence similarity with some high-frequent k-mers. Our experience shows that reads mapping tools work well for this purpose. The main benefit of mapping-based alignment is that it allows small insertions and deletions, and thus can find more repeat-related k-mers. We only consider the lower frequency k-mers that are of intermediate frequency (by default three times or more over the read depth). This not only speeds up the computation and also reduces false positives. This is because k-mers from highly divergent parts of repeats still tend to have frequencies higher than average. BWA “mem” is used with option “-T” to set the minimum score for the alignments. Since we want to avoid false positives, we use no penalty for mismatch, gap open, gap extension and mismatch, and set *k*−5 as the minimum score by default for reads mapping. This step can be performed iteratively if users want to construct more fully constructed repeats. Note that this step may introduce some unrelated k-mers and lead to false positives in repeat assembly. Thus, there is a trade-off in determining how many times this step is run. The mapped k-mers are merged with the highly frequent k-mers and are used as input of the next step.

### K-mer polishing

As illustrated in Fig. 2b, k-mers from repeat copies with variations can often only be assembled to form short contigs. If there are only mismatches on the two k-mers from the same position of two repeat copies, most positions of the two k-mers are still the same. We call these two k-mers “end-to-end” matched. Now suppose there is a single inserted (or deleted) base at the beginning of a k-mer. Then k-mers started from the insertion (or deletion) will be “end-to-end” matched with the k-mer from the other copy that is one base left (or right). “End-to-end” match can be used to generate the consensus k-mers. Consensus k-mers can be more reliable to use for repeat assembly for highly divergent repeats.

Given the merged k-mers (highly frequent and also the mapped intermediate frequent k-mers) generated by mapping-based alignment, a randomized algorithm is used to generate the “end-to-end” matches. For two k-mers with length *k*, we randomly pick *h* bases from the same positions of the two k-mers. If the chosen h-mers are the same, then the two k-mers will be considered as “end-to-end” matched. This procedure runs for *n* times to guarantee the two “end-to-end” matched k-mers are grouped together. Here, we require at least one match between the two h-mers out of *n* times to group the two k-mers. Given the values for *n*,*k*,*h*, the probability *p* of two k-mers being grouped is:

$$p=1- (1-\frac{\binom{k-e}{h}}{\binom{k}{h}})^{n} $$

Here *e* is the allowed edit distance between two “end-to-end” matched k-mers. By default, the value of *e* is set to 1, that is, we allow one mismatch, insertion or deletion in one k-mer. This is reasonable because usually *k* is not large (less than 100).

When matched k-mers are found, we use a weighted voting method for constructing the consensus k-mer. For each position, each k-mer votes for one of the four possible bases with weight *f*, where *f* is the frequency of the k-mer. The base with maximum votes is chosen as the base at that position. The maximum vote out of all positions is considered as the final frequency of the consensus k-mer. This step is implemented in the popular map-reduce way for efficient processing: first we partition the k-mer file into several parts, and then run the polishing step for each part. Finally we merge the results of each partition.

## Results and discussion

To evaluate the performance of the new method, we compare it against the original REPdenovo and RepARK on Human, Arabidopsis thaliana, and Drosophila melanogaster data. These thress species are well studied and have good quality of annotation, which can provide benchmark for our comparison. We use the repeat libraries of these three species released in Repbase [4] as the benchmark. Short sequence reads of one human individual NA19239 from the 1000 Genomes Project [16] is used. The read depth is around 6X with read length 100bp. For Arabidopsis thaliana, the F1 sample released in [17] is used with read depth 10X and read length 250bp. And the Drosophila melanogaster data is downloaded from NCBI (accession number SRR3939094) with read length 151bp and read depth 120X. We compare the divergence rate, copy number and repeat length of the constructed repeat elements. To get the divergence rate and copy number of repeats, we use UCSC annotations [18], which utilizes copy numbers generated by RepeatMasker. We also apply the new method to infer the repeat elements of Hummingbird. There is no existing repeat library for Hummingbird, but there are recently sequenced PacBio long reads [19] which can be used to validate the constructed repeats. For Hummingbird, we use the short sequence reads released in the GeneBank (accession number SRR943146), where the average coverage is around 20X with read length 101bp.

### Comparison with Human, Arabidopsis thaliana and Drosaphila melanogaster data

We evaluate the performance of the two versions of REPdenovo and RepARK by comparing the assembled repeats from these tools with the consensus repeats released in Repbase. There are 1,119, 525 and 238 consensus repeats for Human, Arabidopsis thaliana and Drosaphila melanogaster respectively. In the following, “hits” refers to constructed repeats that are present in Repbase. We use the following metrics previously used in [12] to compare the two versions of REPdenovo to RepARK:

1. The number of Repbase hits with > 85% sequence identity across the length of the Repbase consensus repeat sequence.
2. Average Repbase coverage. For a Repbase hit, this is the average fraction of the Repbase repeat covered by the assembled sequence. We use the set of non-overlapping assembled repeats that achieve the largest coverage.
3. Average Repbase coverage by the longest assembled repeat. One repeat in Repbase may be covered by several constructed repeats. When calculating the average coverage, we choose the longest one.

In Table 1 we show the detailed comparison of the three methods on Human, Arabidopsis thaliana and Drosaphila melanogaster data. Besides the three metrics, we also show the number of repeats in Repbase that are partially (no identity threshold requirement) constructed. The results show that both versions of REPdenovo outperform RepARK on both the number of hit Repbase repeats and the average covered repeat length. In comparison, the original REPdenovo fully constructs 89 (out of the 220 hits), 11 (out of the 68 hits) and 32 (out of the 133 hits) repeats in Repbase for human, Arabidopsis and Drosaphila respectively. And the new version of REPdenovo fully reconstructs 108 (out of the 332 hits), 24 (out of the 102 hits) and 69 (out of the 177 hits) repeats in Repbase for Human, Arabidopsis and Drosaphila respectively. Therefore, our new method significantly outperforms the original REPdenovo in terms of the number of fully constructed repeats.

**Table 1:** Comparison between the two versions of REPdenovo and RepARK on Human, Arabidopsis thaliana, and Drosophila melanogaster data

Species	Methods	*N*	*N*_*h*_	*N*_0_	*C*_*avg*_	*C*_*m*_
	REPdenovo*	6192	108	332	0.61	0.49
	REPdenovo	4648	89	220	0.66	0.55
Human	RepARK	2046	1	168	0.34	0.21
	REPdenovo*	808	24	102	0.42	0.31
	REPdenovo	508	11	68	0.46	0.34
Arabidopsis	RepARK	632	8	59	0.33	0.21
	REPdenovo*	3644	69	177	0.83	0.61
	REPdenovo	3031	33	133	0.67	0.49
Drosophila	RepARK	2,787	26	133	0.66	0.44

REPdenovo*: the new method. N: the total number of repeats constructed. *N*_*h*_ and *N*_0_ are the number of hit Repbase repeats with at least 85% and 0% similarity respectively. *C*_*avg*_: the average Repbase coverage which indicates the average percent of a repeat in Repbase is covered by the constructed repeats. *C*_*m*_: the average Repbase coverage by the longest assembled repeat

Note that for Human and Arabidopsis results, the *C*_*avg*_ and *C*_*m*_ values for the original REPdenovo are slightly larger than the improved version. This is mainly because the new method reports much more repeats than the original REPdenovo. Our experience shows that the new method tends to construct copies of the same repeat with different variations and thus construct more repeats in general than the original REPdenovo. We provide more information in the Conclusions section.

### Comparison between the two versions of REPdenovo

**Fig. 3:**
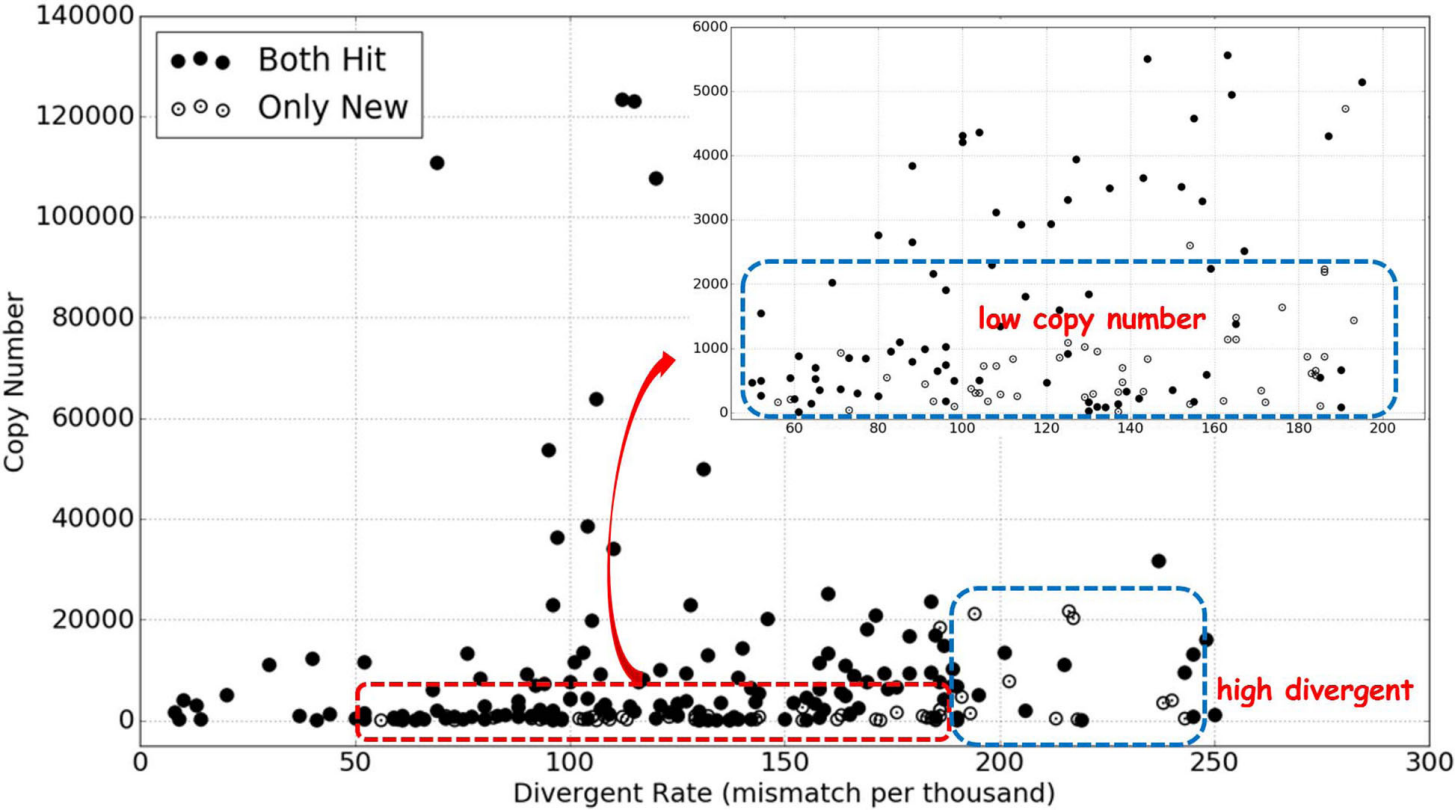
Comparison of the fully constructed repeats in Repbase for the two version of REPdenovo. Bullet circles: hit Repbase repeats constructed by both versions of REPdenovo. Empty circles: hit Repbase repeats constructed only by the new version. Figure in the right-up corner is zoomed in the red rectangle region. There are 154(out of all the 220) bullet circles and 57 empty circles. Most of these 57 ones fall in higher divergent and lower copy number regions (the regions of blue rectangles)

In Section “Method” we show the new method can find more k-mers originated from the repeat regions than the original REPdenovo. As a result, it can construct not only higher diverged regions, but also less frequent repeat elements than the original REPdenovo. As shown in Fig. 3, there are a number of low divergent but low copy number repeats that are only constructed by our new method.

In Fig. 3, we show the comparison between the two versions of REPdenovo on the divergence rate and copy number of the constructed repeats. The bullet circle points are the repeats constructed by both versions, while the empty circle points are the repeats only constructed by the new method. Note that 211 repeats (out of the 332 repeats) are shown in the figure. This is because out of the 332 repeats only 211 can find the divergence rate and copy number information from the UCSC annotations (mainly because the IDs do not match between Repbase and RepeatMasker). The results show that most of the repeats only constructed by the new method have higher divergence rates and are less frequent.

### Repeat elements construction for Hummingbird

**Fig. 4:**
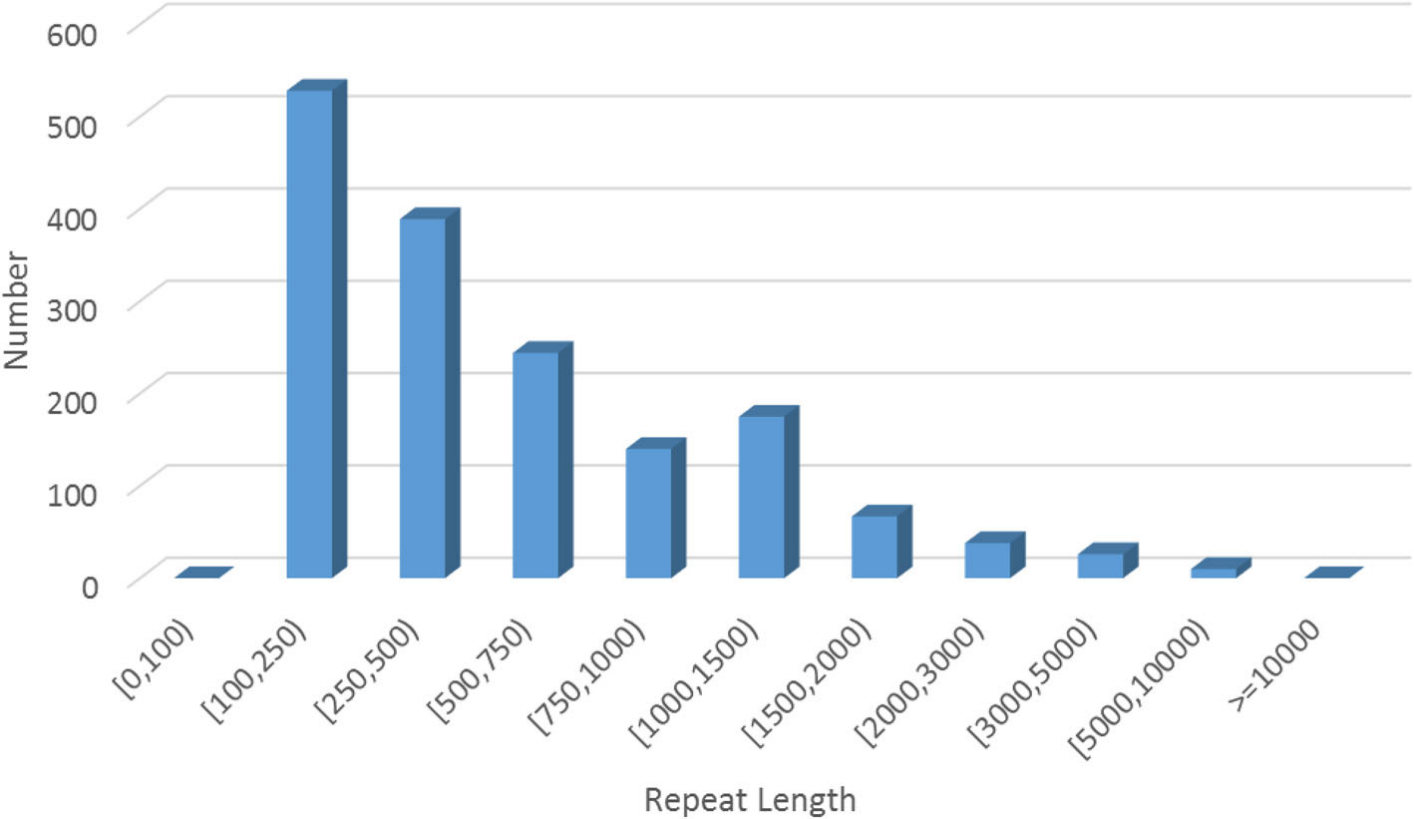
Length distribution of the selected constructed Hummingbird repeats

Our new method can be used to construct repeat elements for species that have no existing repeat libraries or no high quality reference genomes. We apply our new method on the Hummingbird data. 2406 repeats are constructed. Because there is no existing repeat library of Hummingbird to compare with, we cannot directly validate the constructed repeats. Generally, long reads are long enough to cover most of the repeats which provides a way to check whether the assembled repeats are real. Thus, we run NCIB Blast on the constructed repeats to the error-corrected PacBio long reads of Hummingbird. Out of the 2406 repeats, 1617 are almost fully aligned (with similarity larger than 85%). Among these, 1406 are perfectly fully aligned. This indicates that most of the constructed repeats are likely to be real. In Fig. 4 we show the length distribution of the 1617 constructed repeats. Most of the repeats are shorter than 1500bp. There are 64 repeats longer than 2000bp.

To further analyze the constructed repeats, we run RepeatMasker on the 1,617 repeats. In general, RepeatMasker relies on an external repeat library to mask the repeats, which means it will not work for Hummingbird which has no existing repeat library. However, homologous copies of repeats usually exist in multiple species. In this study, we use the “Vertebrate (Other than below)” from RepeatMasker as the DNA source to mask the constructed repeats. Out of the 1,617 repeats 928 are masked and the rest 628 ones are unmasked. Detailed information are shown in Table 2. Note that one repeat may have several regions and the regions may be of different repeat families. Thus one repeat may be reported for several times with different regions and repeat families. For the statistic in Table 2, the row marked as “Unique” only counts those repeats with one unique masked repeat family, while the row marked as “Dup.” allows one repeat counted for more than once. Many of the repeats are masked as “LINE”, which is supported by the known fact that “LINE” repeats widely exist in vertebrate. We believe the 628 unmasked repeats are possibly Hummingbird-only or its close relatives, because they are of high frequency and fully aligned to long reads but have no hits on the “Vertebrate” general library.

**Table 2:** Masking information of the 1617 long reads validated Hummingbird repeats

Category	LINE	SINE	LTR	Retroposon	Satellite	Simple_repeat	Low_complexity	rRNA	Other
Unique	371	0	139	0	10	98	31	0	5
Dup.	557	6	244	0	19	216	52	1	81

For one repeat, RepeatMasker may report several hits depending on whether the repeat is composed of regions of different repeat types. “Unique” only counts those repeats with one unique masked repeat family, while “Dup.” allows one repeat counted more than once

## Conclusion

**Fig. 5:**
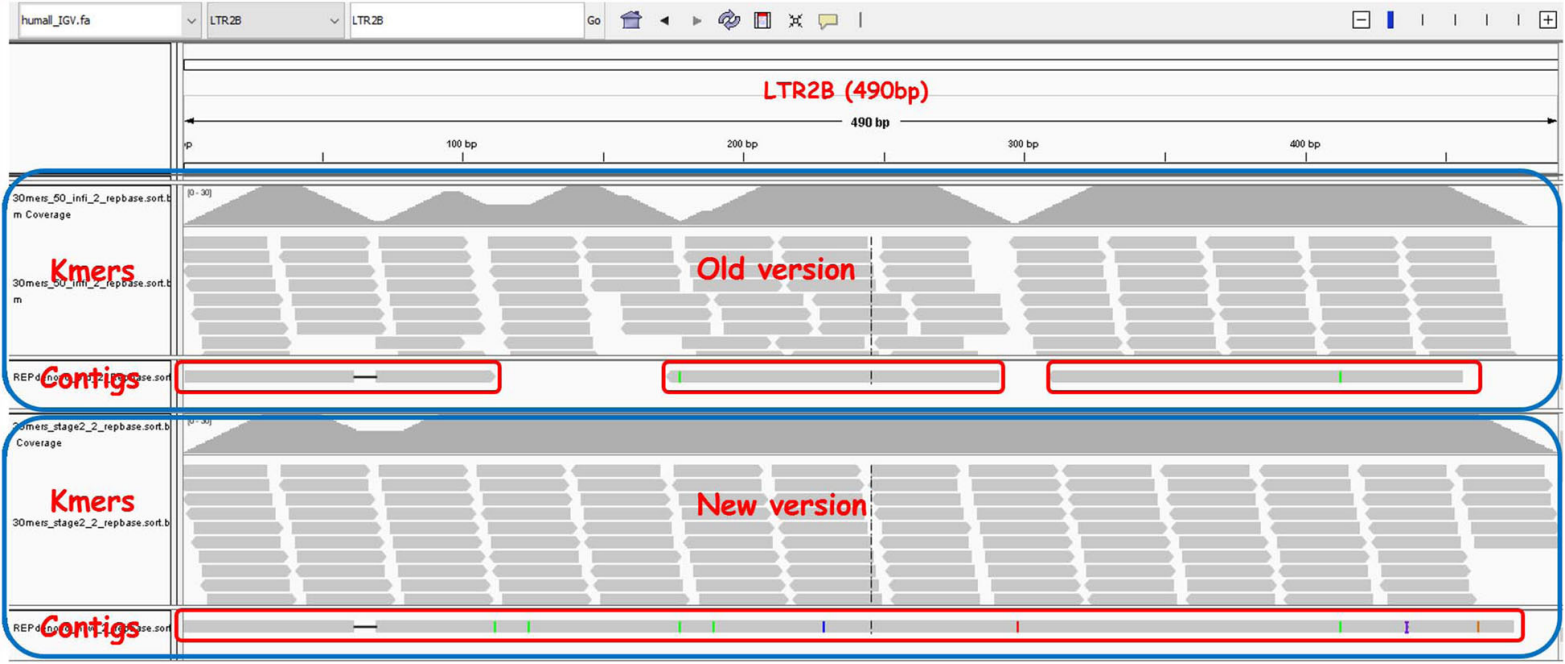
Comparison between the two versions of REPdenovo on constructing one sample repeat “LTR2B”. The old version generates three pieces of the repeat, while the new version constructs the whole repeat

In this paper, we propose an improved method for reconstructing repeat elements directly from short sequence reads. Our new method is able to collect more repeat-related k-mers. Results on both Human, Arabidopsis and Drosaphila data show that the new method can fully construct more repeats in Repbase than the original REPdenovo and RepARK, especially for repeats of higher divergence rates and lower copy number. In Fig. 5, we show the comparison of the two versions of REPdenovo on constructing one sample repeat “LTR2B”, which is mentioned in section 3. The original REPdenovo generates three pieces of the repeat, while the new version constructs the whole repeat.

We also apply the new method on Hummingbird data and assemble 1,619 repeats that can be validated from PacBio long reads. Many of these repeats are likely to be novel (i.e. previously not present in RepBase). We note that long sequence reads (e.g. PacBio reads) may provide new data for repeat analysis. We believe that our method can still be useful for repeat analysis especially for longer repeats even when long reads are available. For example, our method assembles 64 Hummingbird repeats that are longer than 2,000 bp, which can be difficult to analyze even with long reads.

The new method reports more repeats than the original REPdenovo. There are two main reasons for this increase. First, many repeats are of high divergence rate and many constructed contigs are just fragments of one repeat. As more repeat-related k-mers are used in assembly, many previously uncovered regions are constructed, although many are just fragments of the repeat. The other source of more repeats by the new method is that many repeats are just copies of the same repeat consensus. To evaluate how many constructed repeats are from the same repeat consensus, we design the following copy cluster algorithm: First, we check the pairwise similarity between each two repeats, and if the similarity is larger then threshold (by default 0.85), we view the two repeats are of the same group. Then a union find set algorithm is used to cluster the repeats. We apply the clustering on the 6,192 constructed repeats of human individual NA19239, and 3,196 groups are reported. Therefore, the number of constructed repeats can be greatly reduced when related copies are removed.
